# Smartphones and Web 2.0. interventions for weight management

**DOI:** 10.3389/fdgth.2025.1497680

**Published:** 2025-04-10

**Authors:** Muhammad K. Khan, Ambreen Liaqat, Ziyad A. Altokhais, Bader A. Alotaibi, Maryam Sadiq, Munazza Rehman, Zeeshan Ahsan Allana, Hasan N. Tahir

**Affiliations:** ^1^ Department of Community Medicine, College of Medicine, Shaqra University, Shaqra, Saudi Arabia; ^2^ Department of Community Medicine, Holy Family Hospital, Rawalpindi, Pakistan; ^3^ Prosthodontics Department, Dow University of Health Sciences, Karachi, Pakistan; ^4^ Department of Medicine, Aga Khan University, Karachi, Pakistan

**Keywords:** smartphone, Web 2.0, weight management, BMI, waist circumference, intervention

## Abstract

**Introduction:**

This systematic review and meta-analysis examine the effectiveness of smartphone and Web 2.0 interventions for weight management compared to traditional control interventions. The potential of smartphones and Web 2.0. technologies to transform health care and clinical intervention in the community are tremendous. This potential is incredibly increased by increasing adoption rates for smartphones and internet technologies.

**Methodology:**

Ten randomized control trials published between 2015 and 2024 searched through PubMed and ScienceDirect were included. All studies with open access that assessed a smartphone or app intervention compared to a control group in randomized control trials, with weight-related body measures (i.e., body weight, BMI, waist circumference) and physical activity changes (steps/day) expressed in terms of mean and standard deviation performed in a population of adults were included. Review Manager software, version 5.4 (The Nordic Cochrane Centre, The Cochrane Collaboration) was used for statistical analysis.

**Results:**

The results of our study indicate that digital interventions, particularly those utilizing direct communication methods like text messages and social media, significantly promote weight loss and reduce waist circumference (mean difference of −2.12 and −2.81 for weight change and waist circumstances respectively). While reductions in body mass index (BMI) with mean difference of −0.53 were less pronounced, they still favored intervention groups. Subgroup analyses performed to find out the source of heterogeneity revealed that three-arm randomized control trials, studies with larger sample sizes, and interventions lasting around six months showed more consistent and significant effects whereas for sensitivity analysis no significant change in heterogeneity was observed for all parameters. High heterogeneity among studies suggests the need for standardized study designs and intervention protocols in future research.

**Conclusions:**

Despite limitations such as technological issues and engagement variability, these findings underscore the potential of digital health interventions in addressing the global burden of obesity and related non-communicable diseases.

## Introduction

Weight management involves a mix of behaviors, techniques, and bodily processes aimed at helping people reach and maintain a healthy weight (1, 2). The usual approach focuses on achieving a healthy weight through steady, gradual weight loss and then keeping that weight stable. Overweight and obesity are significant global health issues, ranking as the fifth leading cause of death worldwide, with about 3.4 million deaths annually linked to these conditions (3, 4). In 2019, having a higher-than-ideal body mass index (BMI) was estimated to have caused 5 million deaths due to diseases like heart disease, diabetes, cancers, and respiratory and digestive disorders (5). Addressing these issues has become a global health priority. Recent guidelines on preventing and managing non-communicable diseases emphasize the importance of behavioral changes and the need for more user-friendly and effective prevention programs (6).

The rise of smartphones and Web 2.0 technologies holds great promise for transforming healthcare and community-based clinical intervention (7). Numerous studies have explored how smartphones can support health initiatives, such as collecting health data for research and enhancing medical education and clinical practices in the community (8) and as used in support of medical and health care education and clinical practice in the community (9). This potential is incredibly increased by increasing adoption rates for smartphones and internet technologies. The adoption of smartphones and internet technologies continues to grow, with over two-thirds of the world's population using mobile phones. As of April 2024, there were 5.65 billion unique mobile users globally (10). Web 2.0 refers to websites that emphasize user-generated content, ease of use, and interoperability. Social media apps, which are a part of this movement, allow users to create and share content, engaging a large portion of the population (11). Health and fitness apps, in particular, have seen rapid growth. In 2023, these apps generated $3.58 billion in revenue, marking a 9.1% increase from the previous year, with 368 million users (12).

With these trends, research into health interventions using digital technologies is also increasing Studies often look at how these technologies impact general health promotion, including efforts to quit smoking (13) weight management (14) and diet and physical activity or they evaluate the effect on health care delivery programs (15).

### Aims

This review aims to summarize recent eHealth research on using smartphones and Web 2.0 technologies for weight management, comparing them with other interventions, and provide recommendations for future research and practice.

### Objectives

The objectives of this systematic review and meta-analysis are to evaluate the overall effectiveness of smartphone and Web 2.0 interventions in weight management. It seeks to identify the key components of these interventions and analyze the characteristics of the target populations, including demographic and socioeconomic factors. It also targets to determine user adherence and engagement levels, and how these factors influence the success of the interventions. Finally, it aims to highlight gaps in the current literature and propose directions for future research.

## Methodology

### Search strategy

The protocol for this study was registered at PROSPERO and the registration no. is CRD42024556096. A systemic literature search of two databases was conducted through May, 2024 to pick out studies related to the efficacy of smartphone and Web 2.0, intervention compared to a control intervention in achieving body measurement and physical activity changes; Medicine (via PubMed; National Library of Medicine, Bethesda, MD; started in 1966) and ScienceDirect (Elsevier; started in 1997). Search strategy used for data search included the words (mobile phone OR smartphone OR Web 2.0, technologies) AND (weight management). In addition, reference lists from relevant original research and reviews were also reviewed.

### Study selection

**Figure 1:**
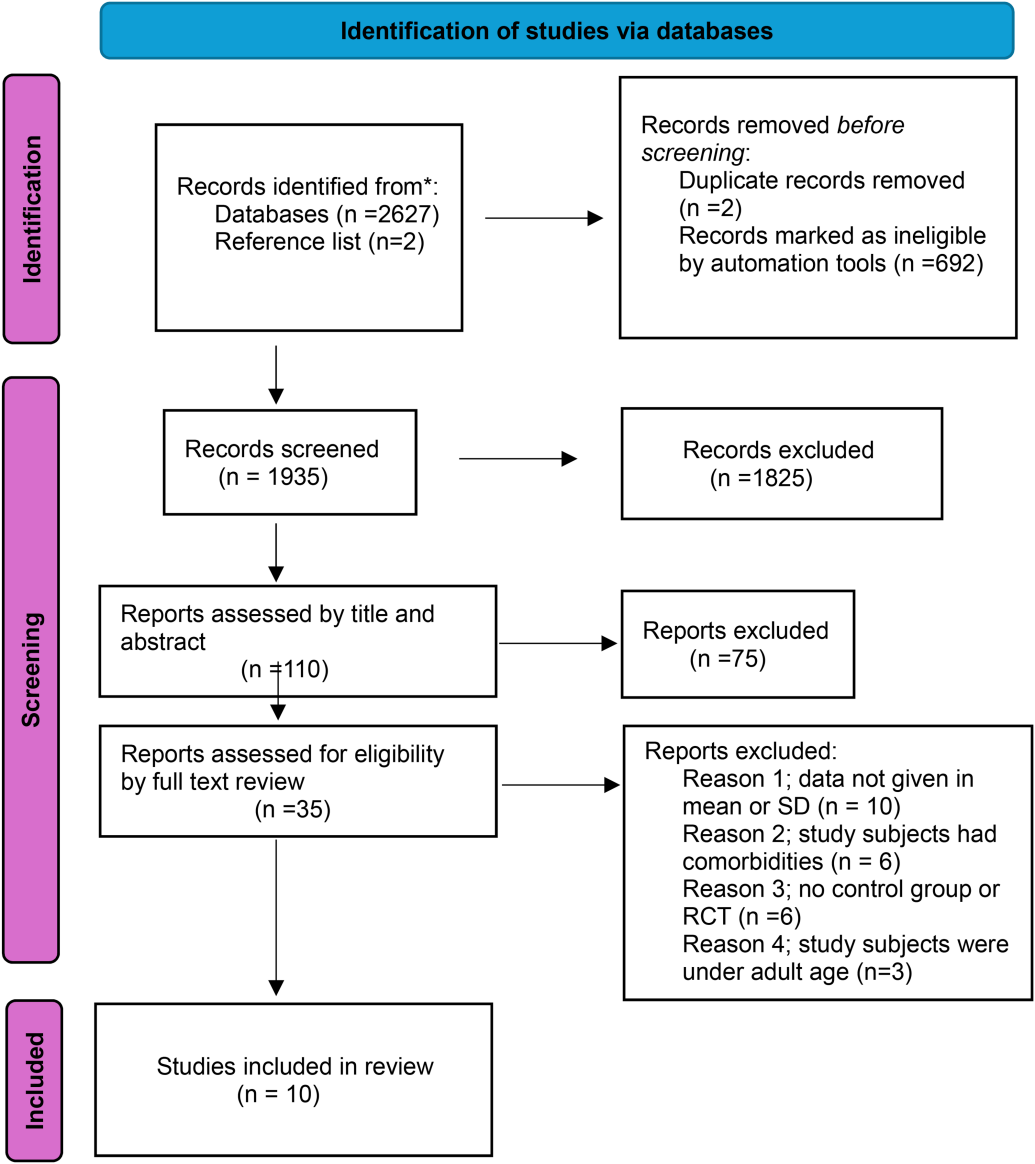
Flow chart for selection of articles.

Studies generated from the databases were screened for inclusion criteria. The study selection process is summarized in Figure 1.

### Inclusion criteria

All studies with open access that assessed a smartphone or app intervention compared to a control group in randomized control trials, with weight-related body measures (i.e., body weight, BMI, waist circumference) and physical activity changes (steps/day) expressed in terms of mean and standard deviation performed in population of adults and published between the years of 2015–20124 were included.

### Exclusion criteria

The exclusion criteria was as follow

1) no original research i.e., reviews, editorials, or non-research letters
2) case reports
3) data on body measures or physical activity not reported or if reported in other then mean terms
4) no control group
5) participants with any disease except a diagnosis of obesity.

### Ethical approval

Ethical approval was not required as only published freely accessed data was analyzed.

### Data extraction and quality assessment

Data was extracted from articles that met the selection criteria and compiled using a table developed in Microsoft Word. Data such as author, year of publication, sample size, intervention, study outcomes etc. was collected. Study outcomes (change in body weight, BMI, waist circumference and physical activity) were recorded in terms of mean and standard deviation. Two studies (16, 17) provided data in terms of percentage which was then converted into gross values using reverse percentage formulas. Four studies (16, 18–20) were three arm RCTs. In such cases the interventional group involving smartphone or web 2.0, technology exclusively was considered for comparison with the control. The risk of bias was assessed using the Cochrane risk of bias tool- robvis, considering random sequence generation, allocation concealment, blinding of participants and personnel, blinding of output assessment, incomplete outcome data and selective reporting (18). Each was categorized as clearly yes, not sure or clearly no.

### Statistical methods

For each study, the net effect size was calculated as the change in body weight measures and physical activity outcomes resulting from intervention from baseline till the end of intervention in the intervention group minus the change in the control group identical measures during the same. For studies (16, 17) providing data in percentages following formulas using baseline values were used to calculate gross value in standard measuring units;

Convert the mean percentage change to a decimal:

Δ=Δ%÷100

Calculate the mean difference in standard units:

ΔW=Δ×W

If there was a need to calculate the standard deviation of the mean percentage difference, it is proportional to the baseline standard deviation:

ΔSD=Δ×SD

If the mean and SD for change between baseline and the end of the intervention were not reported (19), it was calculated using the following equation (20).

For mean;

ΔW=Wpost–Wpre

For SD;

SD2diff=SD2pre+SD2post−2×ρ×SDpre×SDpost

Where SDpre corresponds to the SD at baseline, SDpost corresponds to the SD at the end of interventions, and *ρ* is the correlation coefficient for correlations between measurements taken at baseline and at the end of the intervention assuming it to be perfect i.e., 1.

For body weight, BMI and waist circumference, weighted mean differences were estimated using the random effects models. For physical activity outcomes standardized mean differences were estimated using random effects model. Heterogeneity was quantified with 12 statistics, which describes the proportion of total variation in study estimates as a result of heterogeneity (21). The publication bias was assessed by using Egger's test and funnel plots. Statistical analyses were performed using Review Manager software, version 5.4 (The Nordic Cochrane Centre, The Cochrane Collaboration).

## Results

### Systematic review

The search strategy generated total of 2,629 articles from two different sources. Out of these, 10 articles were included in this meta-analysis (16–20, 24–28). Studies published from 2015 to 2024 were included. All studies included were randomized control trials. The sample size ranges from 30 (19, 22) to 750 (23). The rest of the characteristics of included clinical trials are given in Table 1. Only two studies (19, 22) provided data regarding the change in physical activity in terms of steps/day. For one study the mean change of step/day in intervention group was 7,054.6 whereas in the control group it was 5,002.4. For the second study the intervention group showed a mean change of 736.71 as compared to a control group with mean change of 218.79 in steps/day.

**Table 1:** Characteristics of included clinical trials.

S.No	Author, year	Country	Study design	Population	Sample size	Age mean
1	Kathrin, 2024 (31)	Germany	RCT	Obese men and women	168	46.8
2	Stephan, 2020 (32)	Scotland	Three arm RCT	Men with obesity recruited through community outreach and general practitioner registers	105	52.2
3	Corby 2015 (24)	USA	RCT	Overweight and obese adults	40	44.4
4	Vija, 2017 (33)	Latvia	RCT	Overweight and obese adults	123	36.8
5	Kelly, 2019 (19)	New Mexico	Three arm RCT	Obese adults	30	43.2
6	Nina 2017 (34)	Denmark	RCT	Obese employees in the social welfare and health care sector	369	47.0
7	Monica, 2017 (16)	Australia	Three arm RCT	Overweight and obese individuals	137	50.4
8	Zhang, 2023 (23)	China	Three arm RCT	Elderly overweight and obese	750	79.1
9	Pamela, 2022 (22)	USA	RCT	Older overweight black women	30	65
10	Ingirid, 2018 (17)	Norway	RCT	Physically inactive adults	111	47.85
	Author, year	Duration	Outcome	Intervention	Description of intervention	Control group intervention
1	Kathrin, 2024 (31)	24 weeks	Body weight, body composition and quality of life	App-based intervention	Oviva Direkt fur Adipositas for self-management, self-monitoring and education	Received app-based intervention after 12 weeks of waiting
2	Stephan, 2020 (32)	12 months	Body weight	Narrative text messages plus incentives	Narrative text message library consisting of 604 texts were written by professional script writers €400 incentive at baseline	Received access to the information section of the webpage, printed information and a pedometer
3	Corby 2015 (24)	12 weeks	Body weight, waist circumference, systolic and diastolic blood pressure	Smart loss app	Smart loss provides the ability to deliver intensive behavioral weight loss interventions with monitoring of progress and delivery of personalized treatment recommendations	Received on health tips delivered via text message or emails
4	Vija, 2017 (33)	1 year	Body weight, BMI, waist circumference, hip circumference, waist hip ratio	SMS message once in two weeks	Messages divided into two groups, 1) informative and cognitive 2) encouraging and behavioral	Advice on behavioral lifestyle changes
5	Kelly, 2019 (19)	12 weeks	Body weight, steps/day	Health coaching delivered through videoconferencing and health app	Coaching provided according to the data collected by the withing app, transferred through health app to EMR online database accessible by both researchers and participants	Received only m- health device, no HC session or team member feedback
6	Nina 2017 (34)	38 weeks	Body weight, waist circumference and body fat percentage	SoSu-life web and app-based tool	Tool's features were self-reporting of diet and exercise, personalized feedback, suggestions for activities and practice tips and tricks	Only baseline examination, no intervention
7	Monica, 2017 (16)	24 weeks	Body weight, waist circumference, hip circumference, fasting blood glucose, step count	Weight management program (total wellbeing diet) delivered by social media	Weight management program within a Facebook group along with a support network with the group	Instructed to follow Australian Government dietary guideline as well as the National Physical activity guidelines for adults as Standard care
8	Zhang, 2023 (23)	3 months	Dietary intake, physical activity, indexes of weight control, indexes related to health benefits	Remote management system smartphone app for Dietary and physical activity interventions	The remote management system includes information collection, health assessment, guidance and feedback and follow up	Health education book
9	Pamela, 2022 (22)	12 weeks	Body weight, BMI, waist circumference, physical activity, HbA1c	Text messages plus virtual social support	Physical activity promotion text messages daily and virtual support through Fitbit community created via Fitbit inspire device	1 neutral message related to general health information weekly plus physical activity information booklet
10	Ingirid, 2018 (17)	6 months	Body weight, BMI, waist circumference, fat percentage by skinfolds	Feedback plus recommendations, a leaflet, motivation counseling	Feedback and recommendations for physical activity, leaflets on national dietary recommendations, prompts and reminders, fortnightly motivational counseling via telephone or email	No follow up during intervention period

## Meta-analysis

### Changes in body weight

**Figure 2:**
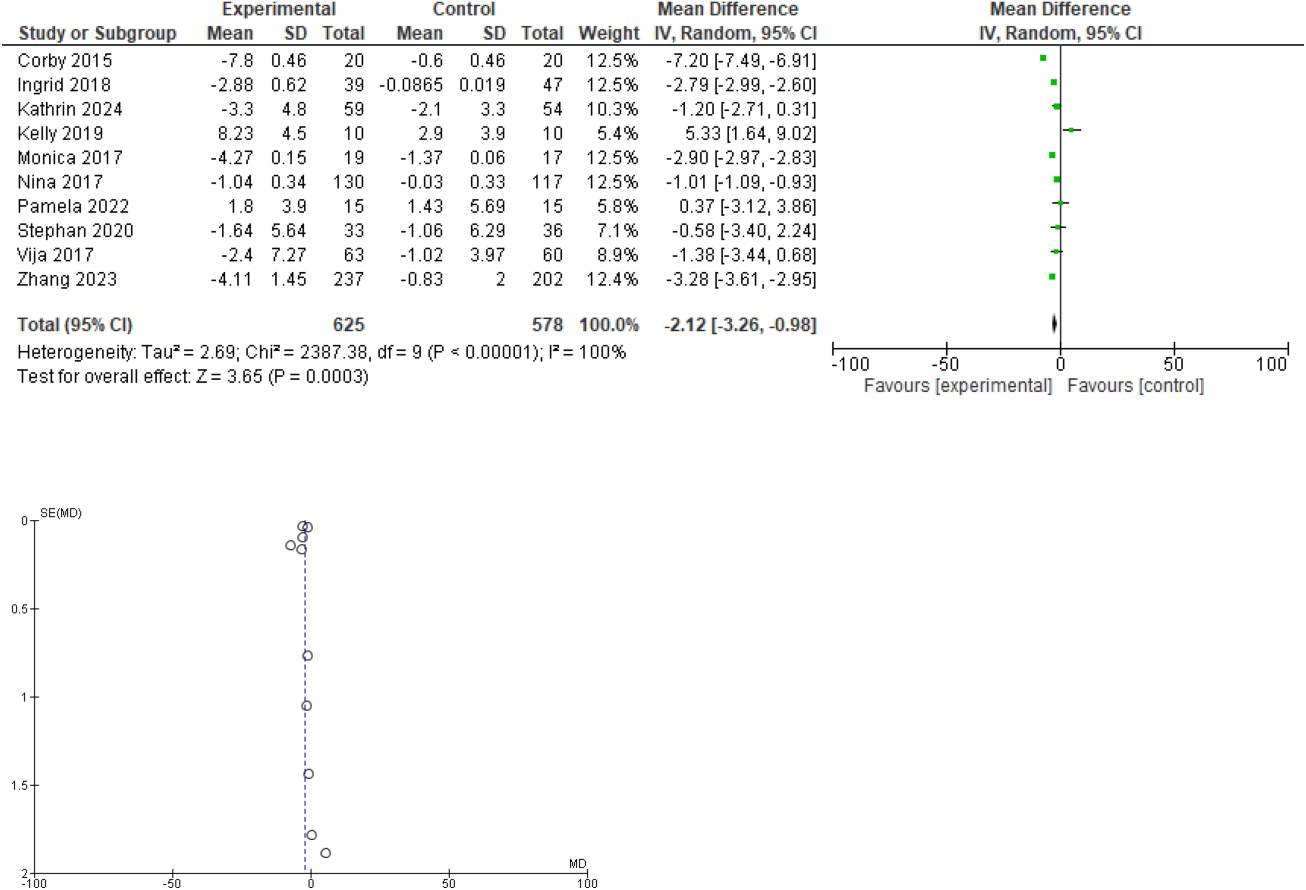
Forest plot for change in weight(kg). Funnel plot for change in body weight.

All 10 researches provided data regarding weight change in the experimental and control groups before and after the intervention. The overall mean difference of −2.12 suggests that, on average, the experimental group performs better than the control group. The high I^2^ value (100%) and significant Chi^2^ test (*P* < 0.00001) indicate substantial heterogeneity among the studies. The overall effect is statistically significant (Z = 3.65, *P* = 0.0003), indicating a significant difference between the experimental and control groups. The funnel plot indicates a low likelihood of publication bias and suggests that the meta-analysis results are robust. The studies are fairly symmetrical around the overall mean difference, with most points close to the vertical line, reinforcing consistency in the effect estimate. Forest plot and funnel plot are given in Figure 2.

### Change in waist circumference

**Figure 3:**
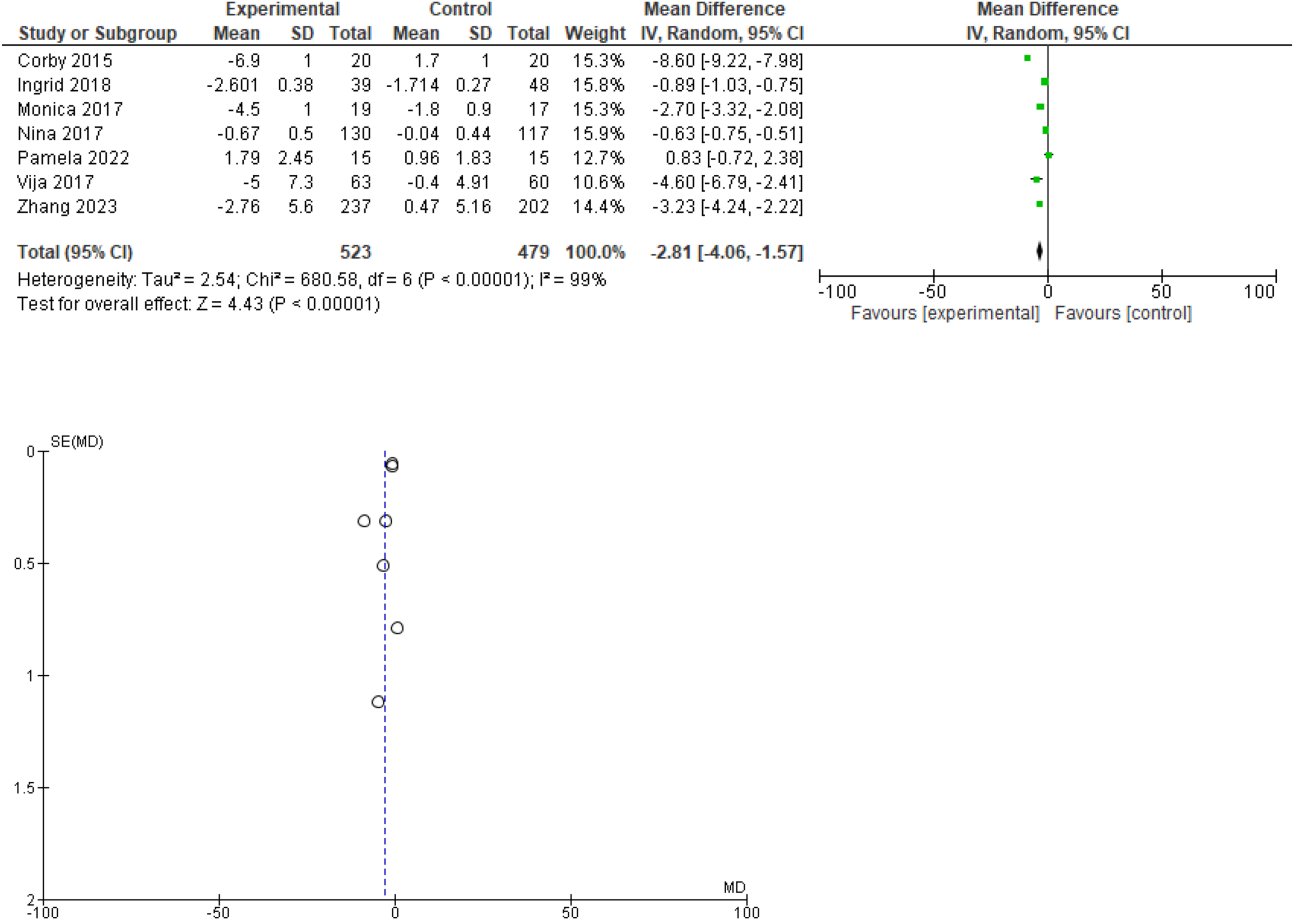
Forest plot for change in waist circumference(cm). Funnel plot for change in waist circumference.

Out of 10, 7 studies provided data regarding waist circumference. The forest plot indicates that the experimental treatment consistently outperforms the control across the included studies, with a combined mean difference of −2.81 95% CI ranging from −4.06 to −1.57. The *Z*-test for the overall effect is 4.43 (*P* < 0.00001), showing a statistically significant overall effect favoring the experimental treatment. The results are statistically significant, despite high heterogeneity, suggesting that the experimental treatment is generally more effective, but the effect size varies between studies. The funnel plot indicates a low likelihood of publication bias. Forest and funnel plots are given in Figure 3.

### Change in BMI

Out of 10, 5 studies provided data regarding BMI. In the forest plot the overall mean difference suggests a small reduction in body weight in the experimental group compared to the control group, but the confidence interval includes zero, indicating that this result is not statistically significant at the 95% confidence level.

**Figure 4:**
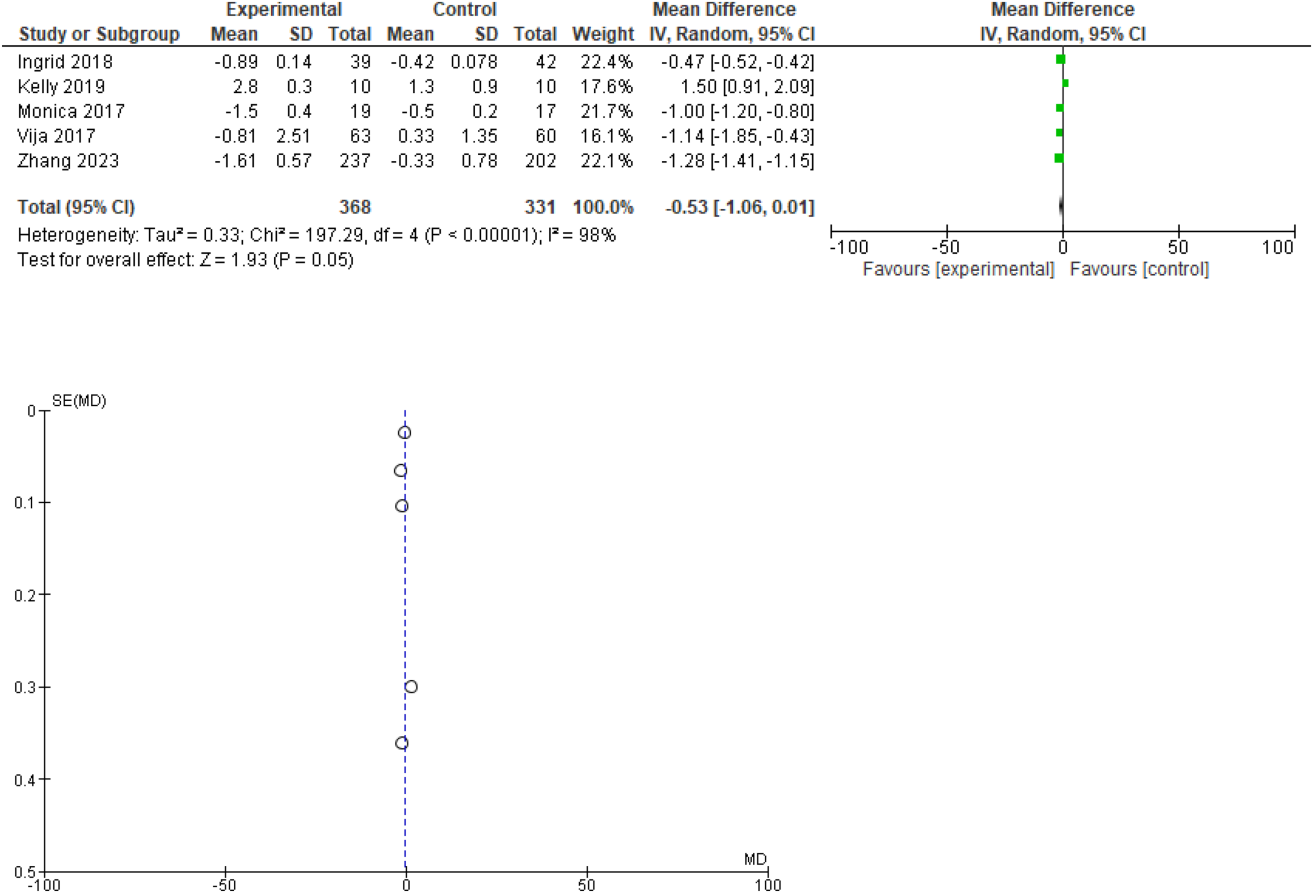
Forest plot for change in BMI (kg/m^2^). Funnel plot for change in BMI.

High heterogeneity (I^2^ = 98%) suggests substantial variability among the study results, which should be explored further to understand the sources of this heterogeneity. The funnel plot indicates a low likelihood of publication bias. Forest and funnel plots are given in Figure 4.

### Sensitivity analysis

To find out the source of high heterogeneity, we performed sensitivity analysis by excluding one study at a time. Sensitivity analysis done for change in body weight produced a variety of values for pooled WMDs ranging from −160 to −2. 55 with the exclusion of (24) producing maximum deviation from original pooled WMDs whereas no significant change in heterogeneity was observed in any case. For change in waist circumference, the range for pooled MDs was −1.56 to −3.35 with heterogeneity not varying significantly. For change in BMI, the range was −0.31 to −0.96 with no significant change in heterogeneity. Sensitivity analysis shows that excluding individual studies does not significantly change the overall heterogeneity. This reinforces that the observed variability is systematic and not due to outliers.

### Subgroups analysis

We also performed subgroup analysis by dividing studies on the basis of study design, sample size, study duration and type of intervention to find out the source of heterogeneity. Details are given in Table 2.

**Table 2:** Subgroup analysis.

Outcomes	Subgroups		
	On the basis of the study design		
	RCTs		Three-arm RCTs
Body weight	WMD = −2. 38,95%CI −4. 62 to −0. 14, I^2^= 100%, Z = 2.08(*p* = 0.04)		WMD = −2. 57,95%CI −3. 35 to−1.78, I^2^ = 89%, Z = 6.93(*p* = 0.00001)
Waist circumference	WMD=2.75,95%CI −4. 24 to −1. 27, I^2^ = 99%, Z = 3.64(*p* = 0.0003)		WMD = −2. 85,95%CI −3. 37 to −2. 32, I^2^ = 0%, Z = 10.56(*p* = 0.00001)
BMI	WMD = −0. 71,95%CI −1. 34 to −0. 08, I^2^ = 71%, Z = 2.21(*p* = 0.03)		WMD = −0. 34,95%CI −1. 20–0.52, I^2^ = 98%, Z = 0.77(*p* = 0.00001)
	On the basis of sample size		
	1–100	100–200	>200
Body weight	WMD = −0. 65, 95%CI −8. 86 to7. 55, I^2^= 97%, Z = 0.16(*p* = 0.88)	WMD = −2. 76,95%CI −3. 03 to −2. 49, I^2^ = 62%, Z = 20.01(*p* = 0.00001)	WMD = −2. 14,95%CI −4. 36 to −0. 09, I^2^ = 99%, Z = 1.88(*p* = 0.06)
	WMD −2. 02,95%CI −3. 45 to −0. 60, 12 = 99%, Z = 2.78(*p* = 0.005)		
Waist circumference	WMD = −3. 91,95%CI −13. 15–5.33, I^2^ = 99%, Z = 0.83(*p* = 0.41)	WMD = −2. 44,95%CI −4. 15 to −0. 74, I^2^ = 95%, Z = 2.81(*p* = 0.005)	WMD = −1. 88,95%CI −4. 43–0.67, I^2^ = 96%, Z = 1.45(*p* = 0.15)
	WMD = −3. 20,95%CI −6.57–0.18, I^2^ = 99%Z = 1.86(*p* = 0.06)		
BMI	Not applicable	WMD = −0. 81,95%CI −1. 28 to −0. 35, I^2^ = 93%, Z = 3.44(*p* = 0.0006)	Not applicable
	WMD = −0. 31,95%CI −0. 91–0.29, I^2^ = 96%, Z = 1.01(*p* = 0.31)		
	On the basis of study duration		
	About 3 months	About 6 months	About 12 months
Body weight	WMD = −1. 87,95%CI −4. 96–1. 22, I^2^= 99%, Z = 1.19(*p* = 0.23)	WMD = −2. 82,95%CI −3. 04 to −2.60, I^2^ = 66%, Z = 25.00(*p* = 0.00001)	WMD = −1. 10,95%CI−2.76–0.56, I^2^ = 0%, Z = 1.30(*p* = 0.19)
Waist circumference	WMD = −3. 70,95%CI −9. 00–1.59, I^2^= 99%, Z = 1.37(*p* = 0.17)	WMD = −1. 77,95%CI −3. 54–0.01, I^2^ = 97%, Z = 1.95(*p* = 0.05)	Not applicable
BMI	WMD = −0. 09,95%CI −2. 63–2.82, I^2^ = 99%, Z = 0.07(*P* = 0.95)	WMD = −0. 73,95%CI −1. 24 to −0. 21, I^2^ = 96%, Z = 2.74(*p* = 0.006)	Not applicable
	On the basis of intervention		
	App-based interventions		Text message, emails, phone calls or social media-based
Body weight	WMD = −1. 81,95%CI−4.90–1.29, I^2^ = 100%, Z = 1.14(*p* = 0.25)		WMD = −2. 89,95%CI −3.05 to −2. 55, I^2^ = 55%, Z = 22.21(*p* = 0.00001)
Waist circumference	WMD = −4. 15,95%CI −9. 69–1.38, I^2^ = 100%, Z = 1.47(*p* = 0.14)		WMD = −1. 69,95%CI −3. 17 to −0. 22, I^2^ = 94%, Z = 2.25(*p* = 0.02)
BMI	WMD=0.09,95%CI −2. 63–2.82, I^2^ = 99%, Z = 0.07(*p* = 0.95)		WMD = −0. 81,95%CI −1. 28 to −0. 35, I^2^ = 93%, Z = 3.44(*p* = 0.0006)

Interventions generally show a significant reduction in body weight and waist circumference, especially in studies with larger sample sizes and those conducted for about 6 months.

For BMI, significant reductions are observed mainly in studies with moderate sample sizes and 6-month duration.

These findings suggest that digital interventions, especially those using more direct communication methods like text messages and emails, can effectively reduce body weight, waist circumference, and BMI over certain periods and sample sizes.

The study design (whether the study is an RCT or a three-arm RCT) and the duration of the study (especially around 6 months) seem to be the primary factors contributing to the variation in the meta-analysis results. These factors likely influence the effectiveness and consistency of the interventions being analyzed.

Different study designs might involve variations in control conditions or additional intervention groups. This can lead to differences in observed effect sizes because the comparison conditions are not uniform across studies. The effectiveness of the intervention might be context-dependent. Differences in how interventions are implemented can cause variation in results.

The length of the study can influence how long participants adhere to the intervention, as well as how sustainable the intervention's effects are. Shorter studies might show initial benefits that don't persist, while longer studies might reveal whether these benefits are maintained or diminish over time.

Future research should consider these sources of heterogeneity, potentially focusing on more standardized study designs and appropriate durations to reduce variability and increase the reliability of findings.

### Risk of bias assessment

**Figure 5:**
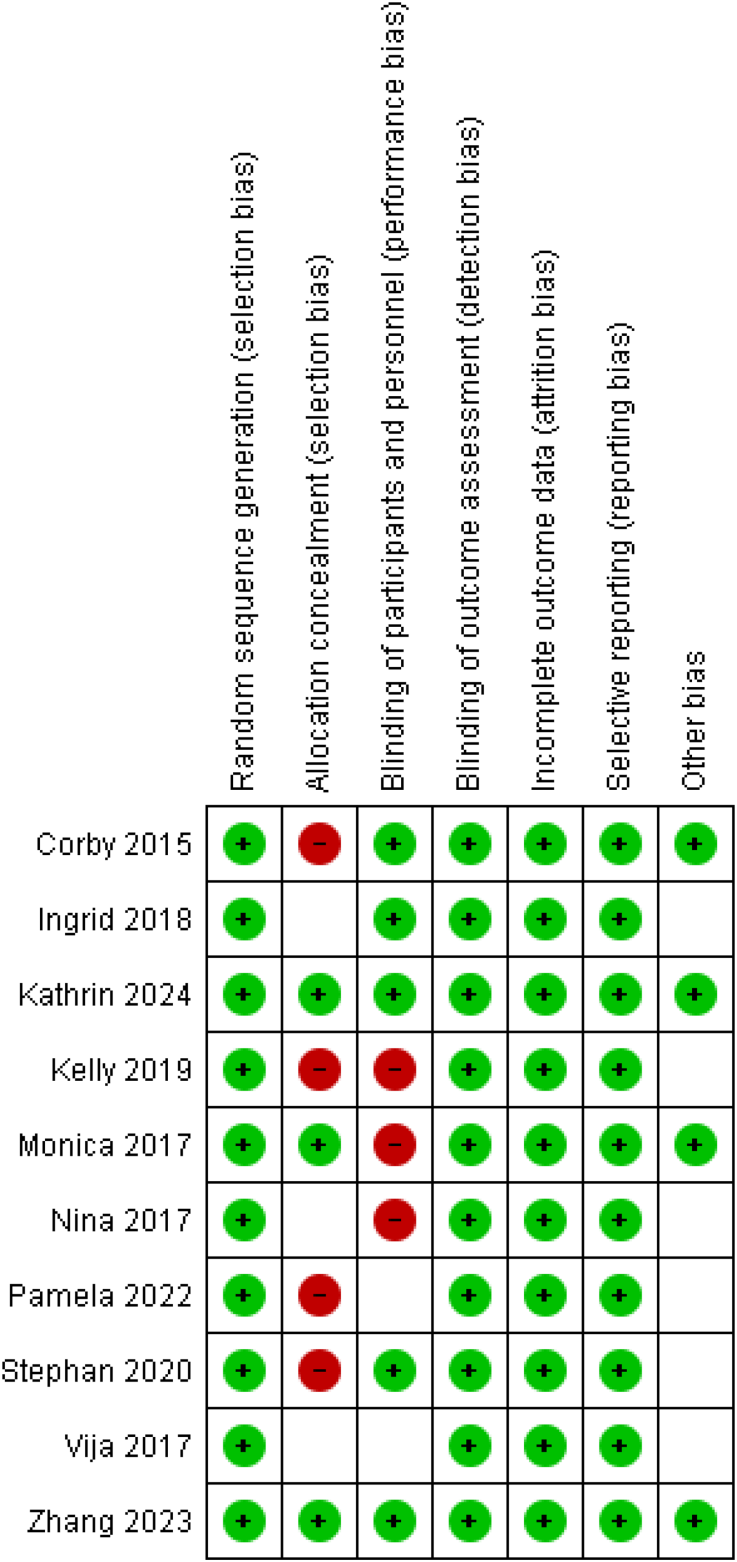
Risk of bias.

The risk of bias assessment for each Cochrane item and each included study in shown in Figure 5. The table indicates that while many studies have strong methodologies in several areas, allocation concealment is a recurring issue. This could affect the overall reliability and validity of the findings in the meta-analysis. Addressing these biases in future research would improve the quality and robustness of evidence. Details are given in Figure 5.

## Discussion

The present systematic review and meta-analysis aimed to evaluate the effectiveness of smartphone and Web 2.0 interventions for weight management compared to traditional control interventions. The findings indicate that digital interventions, especially those employing direct communication methods like text messages, emails, or social media, have significant potential in promoting weight loss and reducing waist circumference. These results are consistent with previous studies that have underscored the utility of digital health technologies in weight management and other health behavior changes. One of the previous meta-analyses suggested that mobile phone app interventions compared with various control interventions significantly reduced body weight by 1.04 kg, reduced BMI by 0.43 kg/m^2^, and non-significantly increased physical activity by an SMD of 0.40 (25). While these reductions in body weight and BMI are modest, it is unrealistic to expect that a single intervention, like mobile apps, would result in significant weight loss compared to other control methods (26).

Significant research in mobile interventions has particularly concentrated on text-messaging, or SMS-based, interventions. A prior meta-analysis (27) revealed that mobile phone interventions led to notable reductions in body weight and BMI compared to control groups, showing decreases of −1.44 kg and −0.24 units, respectively. Another systematic review provided strong evidence from RCTs that mobile technology interventions promote short-term weight loss (28). Additionally, a systematic review encompassing seven studies highlighted the positive effects of text messaging or mobile apps in reducing physical inactivity and overweight/obesity (29). However, another review indicated that many weight-loss apps yield inconsistent results (30).

This meta-analysis revealed a statistically significant reduction in body weight in the intervention groups compared to the control groups, with a pooled weighted mean difference (WMD) of −2.12 kg. This finding aligns with existing literature suggesting that digital interventions can effectively support weight loss efforts through mechanisms such as self-monitoring, feedback, and social support. The reduction in waist circumference was also notable, with a pooled WMD of −2.81 cm, reinforcing the beneficial impact of these interventions on abdominal obesity, which is a crucial marker for metabolic health.

While the reduction in BMI was less pronounced and not always statistically significant, the trend was still favorable toward the intervention groups. This could be due to the heterogeneity of the studies included, as well as variations in the duration and intensity of the interventions. Only two studies provided data on physical activity changes, indicating a significant increase in steps per day among intervention participants, which is consistent with findings from other research emphasizing the role of physical activity in weight management.

High heterogeneity was observed in the meta-analyses of body weight, waist circumference, and BMI, which was not substantially reduced by sensitivity analysis. This suggests that the variability in study designs, intervention types, sample sizes, and durations significantly influenced the outcomes. Subgroup analyses indicated that three-arm RCTs, studies with larger sample sizes, and interventions lasting around six months showed more consistent and significant effects. This finding highlights the importance of considering study design and duration in future research to enhance the reliability and applicability of results. The risk of bias assessment indicated that while many studies had strong methodologies in several areas, allocation concealment was a recurring issue, which could affect the overall reliability of the findings. Ensuring robust allocation concealment in future studies would help improve the quality and robustness of evidence.

Mobile text messaging, phone calls and support through social media play a crucial role in weight management by providing continuous support, motivation, and information to individuals. These interventions leverage the ubiquity and convenience of mobile phones to deliver personalized reminders, tips, and encouragement, which can enhance adherence to dietary and physical activity recommendations. Text messages can also facilitate behavior change by promoting self-monitoring and goal setting, both of which are key components of successful weight management. Additionally, the interactive nature of SMS allows for timely feedback and social support, which are important for maintaining motivation and overcoming obstacles.

Mobile apps that are well-designed hold the promise of transforming health interventions by utilizing technology to reach populations in unprecedented ways, leveraging the unique features of smartphone software. This rapid market growth raises the issue of regulation, especially with the increasing number of advertising claims regarding their efficacy. Researchers are calling for more studies to provide robust scientific evidence on the actual effectiveness of these apps. The constant accessibility of mobile phones ensures that users can manage and reinforce healthy behaviors at any time through a range of communications and applications. These fitness and weight-loss apps allow users to monitor their diet, weight and physical activity, make healthier grocery and restaurant choices, prepare nutritious meals (29) and participate in health interventions (35). Furthermore, users do not need additional devices like pedometers to keep track of their physical activity.

Mobile apps, text messages, social media support, and phone calls for weight management have several limitations. Technological issues, such as app compatibility and functionality, can hinder user experience. Privacy concerns arise due to the sensitive nature of health data. Engagement levels vary, with some users losing interest over time. Personalized feedback is often limited, reducing the effectiveness of interventions. Information overload from constant notifications can be overwhelming. Additionally, accessibility issues affect those who are less tech-savvy or economically disadvantaged. Lastly, the overall effectiveness of these interventions varies, often needing to be combined with other strategies for optimal results.

The results of this meta-analysis underscore the potential of digital health interventions in weight management. However, future research should aim to standardize study designs and intervention protocols to reduce heterogeneity and improve comparability. Moreover, longer follow-up periods are necessary to assess the sustainability of the observed benefits. Addressing methodological issues such as allocation concealment and ensuring rigorous randomization processes will also enhance the quality of future research.

### Limitations

This study's limitations include high heterogeneity among the included trials, which indicates variability in study designs and intervention protocols that could affect the consistency of the results. Additionally, the reliance on self-reported data for physical activity and body measurements may introduce reporting biases. The exclusion of non-English studies and the limited number of trials available for certain outcomes may also affect the generalizability of the findings. Lastly, potential technological issues and variability in user engagement with digital interventions were not thoroughly explored.

## Conclusion

In conclusion, this systematic review and meta-analysis provide robust evidence supporting the use of smartphone and Web 2.0 technologies in weight management interventions. These findings have important implications for public health strategies aimed at addressing the global burden of obesity and related non-communicable diseases.
